# Solvothermal synthesis of facet-dependent BiVO_4_ photocatalyst with enhanced visible-light-driven photocatalytic degradation of organic pollutant: assessment of toxicity by zebrafish embryo

**DOI:** 10.1038/s41598-020-69706-4

**Published:** 2020-08-03

**Authors:** Ganesh S. Kamble, Yong-Chien Ling

**Affiliations:** 1 Department of Chemistry, National Tsing Hua University, Hsinchu, 30013 Taiwan; 2 Department of Engineering Chemistry, Kolhapur Institute of Technology’s College of Engineering (Autonomous), Kolhapur, 416234 India; 3 Institute of Nano Engineering and Micro Systems, National Tsing Hua University, Hsinchu, 30013 Taiwan

**Keywords:** Chemistry, Materials science, Nanoscience and technology

## Abstract

The BiVO_4_ photocatalyst plays a very important role in photocatalytic reactions attributed to its unique crystalline structure, size, morphology and surface area. Herein, we report a facet-dependent monoclinic scheelite BiVO_4_ (m-BiVO_4_) photocatalyst with uniform truncated square (18 sided) hexagonal bipyramidal shape synthesized by a template-free and surfactant-free solvothermal method using ethylene glycol solvent under cost-effective and mild reactions. The structural, morphological and optical properties of the m-BiVO_4_ photocatalyst are widely characterized. The photocatalytic activity of the m-BiVO_4_ photocatalyst is tested towards 20 ppm methylene blue (MB) dye aqueous solution as a pollutant model under visible light irradiation. Enhanced visible-light driven photoactivity with dye degradation efficiency of approx. 91% at a rate of 0.388 × 10^−2^ min^−1^ is obtained, presumably due to the presence of high-active (040) facets. Zebrafish embryo toxicity test of treated MB dye solution reveals the degradation and toxicity reduction of the MB dye. Moreover, the recycling experiment validates that the m-BiVO_4_ photocatalyst has a great structural stability with reliable performance. This work may provide a lucid and expedient strategy to synthesize highly crystalline (040) facet-dependent semiconductor photocatalyst toward dye degradation and obviously industrial wastewater remediation.

## Introduction

Earth is gifted with abundant quantity of clean water. But now days, the world is found in serious risk on environmental and economic stability due to the enormous population and industrialization growth^1^. Today in many industries, organic dyes are used extensively and mainly in textile, leather, cosmetics, plastic, paper, ink, ceramic and food processings. Along with these, huge amounts of industrial effluents containing various organic pollutants such as agrochemicals, drugs and antibiotics have also been discharged into fresh water^2,3^. Particularly in textile industries, dyes are commonly used owing to their favourable characteristics such as water-soluble, low-price, bright colours and easier to apply on the fabric. The dyes are usually categorized based on their chromophoric structures and different types of dyes such as azo, diazo, xanthene, anthraquinone, acidic and basic dyes are readily available. The azo dyes (–N=N–) are most commonly employed in textile industries and exhibit environmental toxicity effects including carcinogenic and mutagenic events to human beings, animals and water bodies^4^. Prior to 2018, it was projected that approximately 70% of the industrially wastewater was not well treated and their discharges causing severe pollution towards natural water bodies. Therefore, it is expected that up to 50% of the people will face clean water disasters by 2025^5^.

Recognizing the potential water pollution issues, worldwide researchers have dedicated their prime efforts to prevent these overwhelming matters by developing highly advanced environmental technologies for a sustainable future^6,7^. The imperative task of removing these pollutants from industrial effluents before their discharge into environment is a remarkable challenge globewise^8–10^. Apart from this, the chemical analysis of target pollutants and degradation products in the industrial effluents and treated wastewater is also important to check the treatment efficiency and environment safety. Additional studies to check the toxicity and quality of the treated wastewater by toxicity test is therefore becoming important. This is confirmed by aquatic organism test. Currently, the aqueous toxicity test as well as the survival analysis are carried out using various aquatic species such as Daphnia, crustaceans, fish, zebrafish embryo, algae, bacterial cells and plants^11^.

Many organic pollutants/dyes cannot be effectively eliminated by the conventional wastewater treatment processes such as biological, physical adsorption, chemical oxidation, and coagulation due to their low efficiency, complicated aromatic molecular structures and secondary pollutants. Among these methods, photocatalytic degradation/adsorption process has become promising for eliminating dyes due to its low-cost, simple design, efficiency, wide adaptability, easy operation and relatively low sludge production^12–15^. In recent years, semiconductor photocatalyst is regarded as a promising tool for remediation of water pollution^16^ and many attempts focused on the development of novel visible-light-driven photocatalysts and their activities studies have been reported^17–22^. Among these, TiO_2_^19,20^, and ZnO^21,22^ are the most popular semiconducting metal oxide photocatalysts used for degradation of organic pollutants in wastewater. Semiconducting photocatalysts have wide band gap energy of 3.0–3.2 eV for UV absorption which accounts only 4% in solar spectrum. Many researchers have therefore becoming interested to synthesize visible-light-driven (VLD) photocatalysts as the solar spectrum embedding 45% visible light. Additional advantages of lower cost and safety use further drive VLD photocatalyst with higher efficiency and practical application feasibility^23,24^. In concern with these issues, monoclinic bismuth vanadates (BiVO_4_) have riveted strong attentions recently owing to its *n*-type metal oxide semiconductor nature^25^, a reliable VLD photocatalyst as a potential photoanode for oxygen (O_2_) evolution reaction through water-splitting. This is attributed to its promising band-gap energy (Eg 2.3–2.4 eV), high optical absorption, nontoxicity and reasonable band edge alignment for redox reactions^26^. Furthermore, its semiconducting nature enable BiVO_4_ absorbing appropriate electromagnetic radiation and inducing the charges by means of produces free electrons (*e*^−^) and holes (*h*^+^) in its conduction band (CB) and valence band (VB), respectively. Both *e*^−^ and *h*^+^ species are very important for the oxidation (*h*^+^) or reduction (*e*^−^) reactions of organic pollutants whilst the pollutants were adsorbed onto the surface of the semiconducting photocatalyst. The same semiconducting photocatalyst may therefore be used for water remediation in industrial scale^27^.

BiVO_4_ exists mainly in three crystalline phases: monoclinic scheelite (m-BiVO_4_), tetragonal zircon and tetragonal scheelite^28^. Among the scheelite compounds, the m-BiVO_4_ (2.4 eV) has relatively smaller band gap energy than tetragonal zircon*-*BiVO_4_ (2.9 eV), which accounts for higher photon absorption properties found in m-BiVO_4_^29^, presumable due to its more VLD activity. The Bi–O dodecahedra are more distorted in m-BiVO_4_ due to a 6s^2^ lone pair of Bi^3+^ also causes its higher VLD activity. The m-BiVO_4_ is a member of aurivillius family of layered oxides known for terroelasticity property and have been extensively provoked in photocatalytic activity. The m-BiVO_4_ photocatalyst is therefore highly preferred for VLD dye degradation purpose. The BiVO_4_ plays a very important role in photocatalytic reactions attributed to its unique crystalline structure, size, morphology and surface area^30,31^. The multifaceted monoclinic BiVO_4_ structures have been synthesized by various methods such as hydrothermal method^32^, chemical bath deposition^33^, co-precipitation^34^, sonochemical method^35^ and organic–inorganic precursor method^36^. Hydrothermal method is one of the well-organized chemical methods for the synthesis of BiVO_4_ with uttered morphology.

Great progress in current hydrothermal synthesis of BiVO_4_ photocatalyst has been achieved on its different shape structures. However, the drawbacks of tedious and costive synthesis step as well as the use of toxic templates and surfactants cannot be overlooked. The developed methods still allow for further improvements (Supplementary Introduction S1 and Table S1) owing to the use of precious and f-block doping elements, critical oxide composite complex, templates and reduced graphene oxides, the appearance of ununiformed morphology of oxide heterojunction catalyst, formation of agglomeration, multiple synthesis steps, Use of various solvents in large quantity, long synthesis time and intensive energy use.

Herein, we have developed a template- and surfactant-free monoclinic m-BiVO_4_ by a simple solvothermal method using ethylene glycol solvent under mild reaction conditions at modest cost and feasible scalability. The highlights are: (a) Facile solvothermal synthesis of (040) facet-dependent BiVO_4_ photocatalyst, (b) Enhanced photocatalytic activities for aqueous MB degradation under visible-light irradiation, (c) The Zebrafish embryo toxicity test confirms the toxicity reduction and mineralization efficiency for MB, (d) Feasible and scalable implementation owing to lower photocatalyst preparation cost and the use of freely accessible sunlight.

## Results and discussion

### Structural, optical, surface and morphological properties of m-BiVO_4_

**Figure 1:**
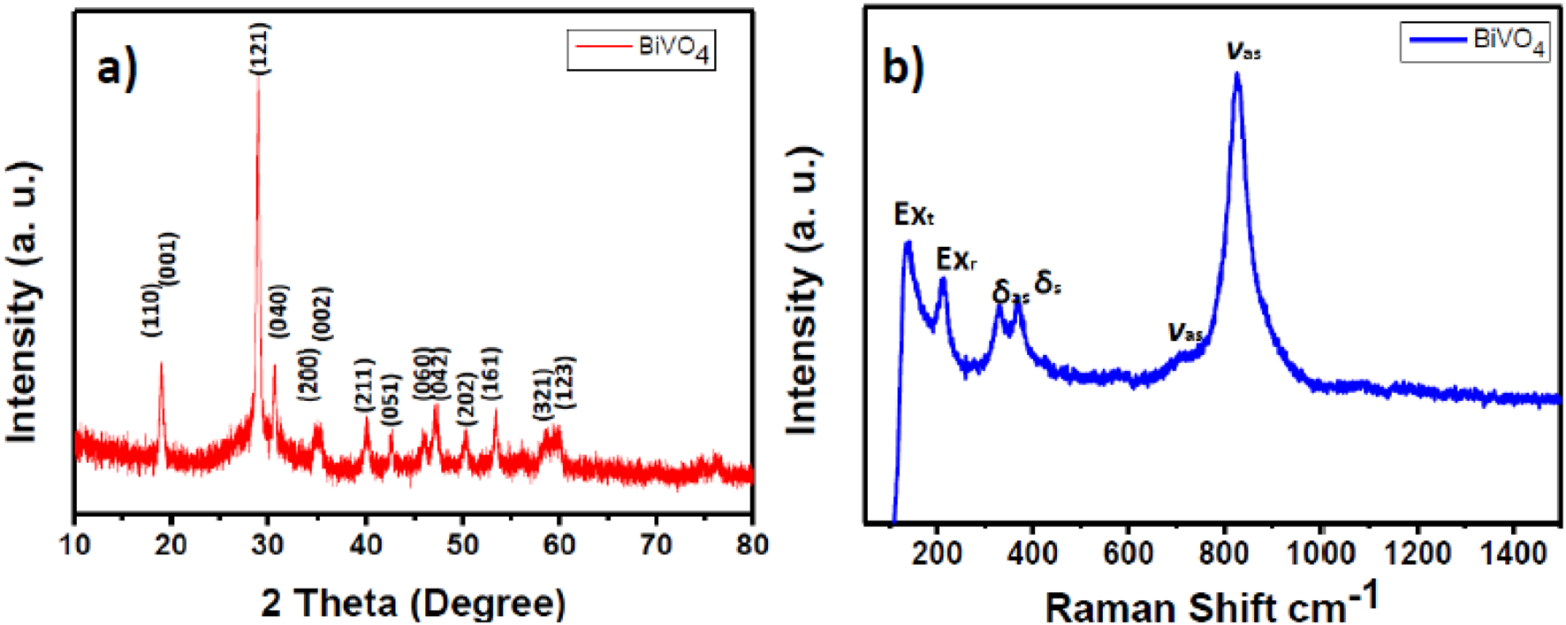
(**a**) XRD patterns and (**b**) Raman spectra of the m-BiVO_4_ photocatalyst synthesized at 250 °C and excited by a red-line laser (632.8 nm).

The crystalline structure of m-BiVO_4_ was characterized by X-ray diffraction (XRD) measurement. The XRD pattern (Fig. 1a) reveals the formation of monoclinic scheelite BiVO_4_ with lattice parameters a = 0.5205 ± 0.0017 nm, b = 1.1721 ± 0.0021 nm, and c = 0.5019 ± 0.0016 nm, which are in good agreement with the reported values a = 0.5195 nm, b = 1.1700 nm, and c = 0.5092 nm (JCPDS card No. 014–0,688). The m-BiVO_4_ is pure monoclinic with hexagonal structure as confirmed by the splitting of the diffraction peaks at 2θ = 18.66°, 18.67°, 29.28°, 30.64°, 35.30°, 35.34°, 40.02°, 42.25°, 46.94°, 47.03°, 50.20°, 53.46°, and 59.80°, which can be indexed to (110), (001), (121), (040), (200), (002), (211), (051), (060), (042), (202), (161), and (321), respectively. The hexagonal truncated structure of BiVO_4_ is known to provide efficient active sites for photocatalysis under solar visible light irradiation^37^.

Raman spectroscopy is more sensitive to provide structural information, degree of crystallinity, defects and disorders, particle size and electronic properties of nanomaterials. The Raman spectrum of m-BiVO_4_ (Fig. 1b) excited by a red laser (632.8 nm) delineates the translational (Ex_t_) and rotational (Ex_r_) phonon vibration bands at 136 and 213 cm^-1^, respectively. The peaks at 328 and 367 cm^−1^are attributed to the typical antisymmetric (δ_as_) and symmetric (δ_s_) bending modes of the vanadate tetrahedral anion. The weak peak at 709 cm^−1^ and dominated peak at 826 cm^−1^ are assigned to the antisymmetric (ν_as_) and symmetric (ν_s_) V–O stretching vibrations in vanadate tetrahedral anion in monoclinic scheelite BiVO_4_^38^.

**Figure 2:**
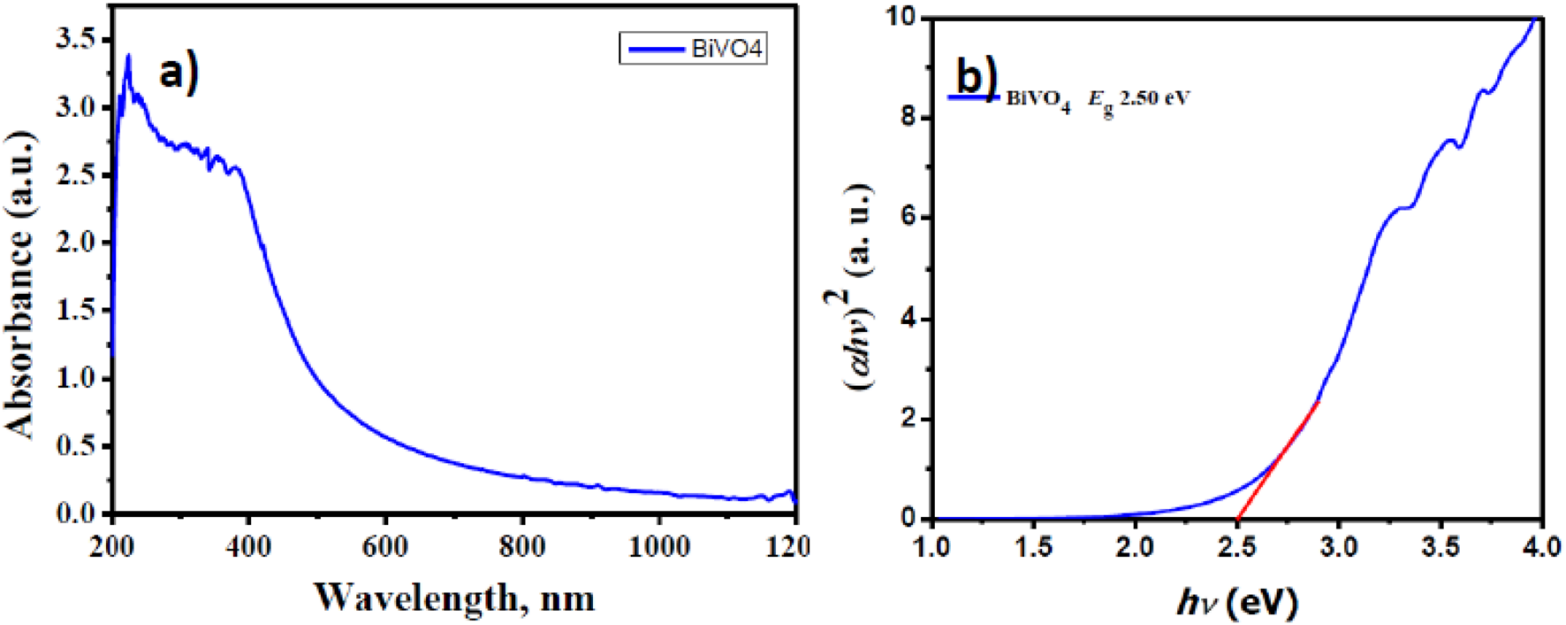
(**a**) UV–Vis absorption spectra and (**b**) Tauc plots of the (*αhv*)^2^ vs. photon energy (*hv*) of the m-BiVO_4_ photocatalyst at room temperature.

The UV–Vis absorption spectrum of m-BiVO_4_ (Fig. 2a) exhibits a strong optical absorption in 420–800 nm wavelength range, indicating that m-BiVO_4_ can efficiently absorb visible light and acts as solar light driven active photocatalyst for dye degradation. It is well known that semiconducting electronic structure usually plays a critical role in its photocatalytic activity. Usually, the valence band (VB) and the conduction band (CB) of BiVO_4_ is poised by hybridized Bi 6s/O 2p orbitals and V 3d orbitals, respectively^30^. The band gap and absorption coefficient according to the Kubelka–Munk equation can be expressed as *αhv* = A(*hv* − *Eg*)^1/2^, where *α, v* and *h* represents the absorption coefficient, frequency of the light and Planck’s constant, respectively. The band gap energy value can be estimated from the Tauc plot (Fig. 2b) of (*αhv*)^2^ versus photon energy (*hv*) curve. The intercept of the tangent to the X-axis is a good estimate of the band gap E_g_ and matches with previous literature^31^. The estimated band gap E_g_ of m-BiVO_4_ is 2.5 eV, confirming its capability as visible light driven (VLD) photocatalyst.

**Figure 3:**
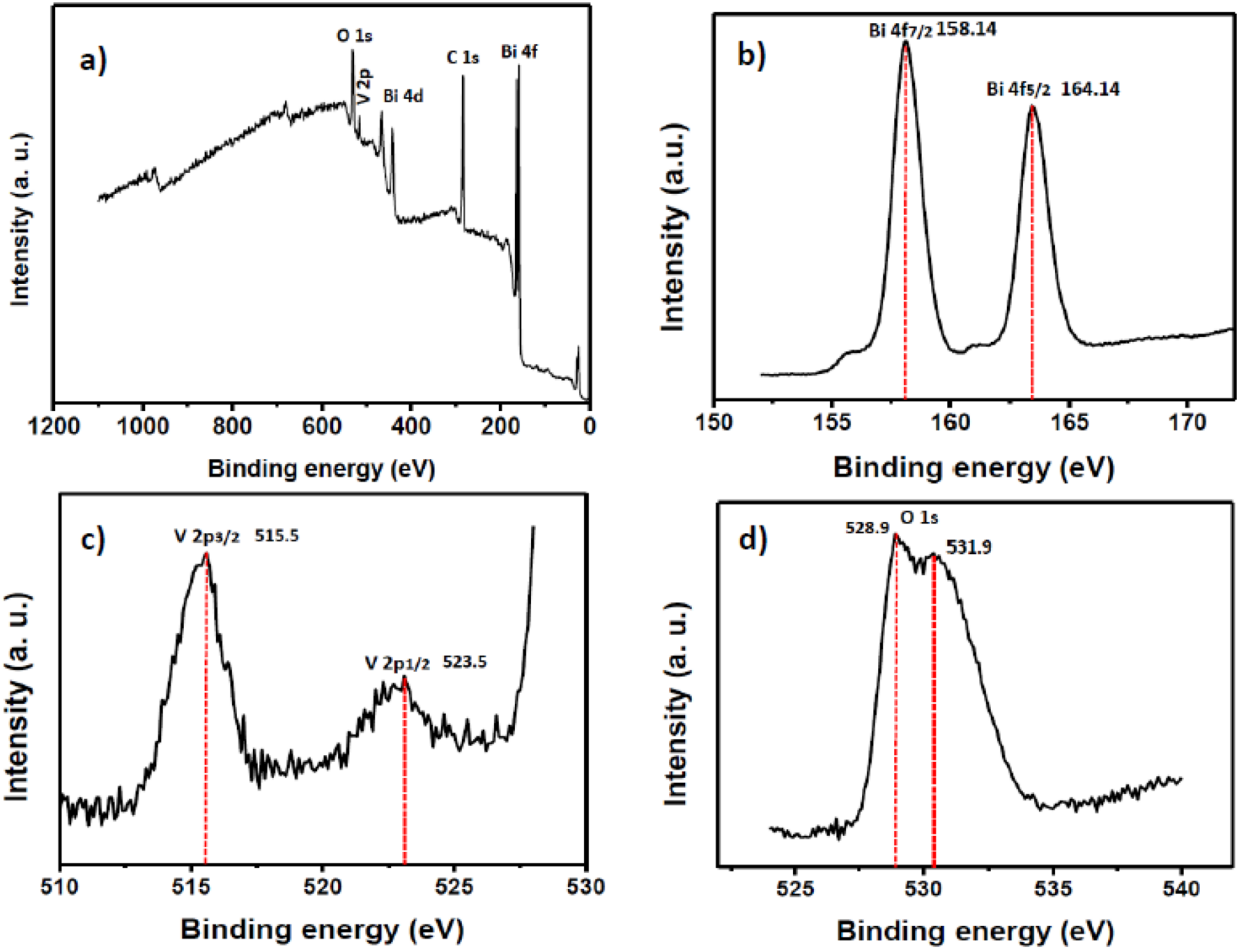
High resolution XPS spectra of (**a**) m-BiVO_4_, (**b**) Bi 4f, (**c**) V 2p and (**d**) O1s of the m-BiVO_4_ photocatalyst.

The X-ray photoelectron spectroscopy (XPS) is used to determine the surface chemical composition of the m-BiVO_4_. The XPS is capable of differentiating the spin–orbit splitting of metal ions at two possible states, i.e. having different binding energies, and provides metal speciation information. The survey XPS spectrum of the BiVO_4_ (Fig. 3a) reveals the presence of C, Bi, V and O elements in the m-BiVO_4_. The high resolution XPS spectrum of the Bi4f spectra (Fig. 3b) exhibits doublets at 158.14 and 164.14 eV that correspond to the Bi4f_7/2_ and Bi4f_5/2_ lines, respectively. The 6.0 eV difference between the two bonding energy suggests that Bi is in a + 3 oxidation state^39^. Whereas, the split peaks of V2p at 515.5 eV and 523.5 eV (Fig. 3c) correspond to V2p_3/2_ and V2p_1/2_, respectively, suggesting the presence of V + 5 oxidation state^40^ in the m-BiVO_4_. The split peaks of O1s at 528.9 eV and 531.9 eV (Fig. 3d) represent an asymmetric behaviour of the oxygen states that can be consigned to the lattice oxygen and surface hydroxyl groups in crystalline m-BiVO_4_, respectively.

**Figure 4:**
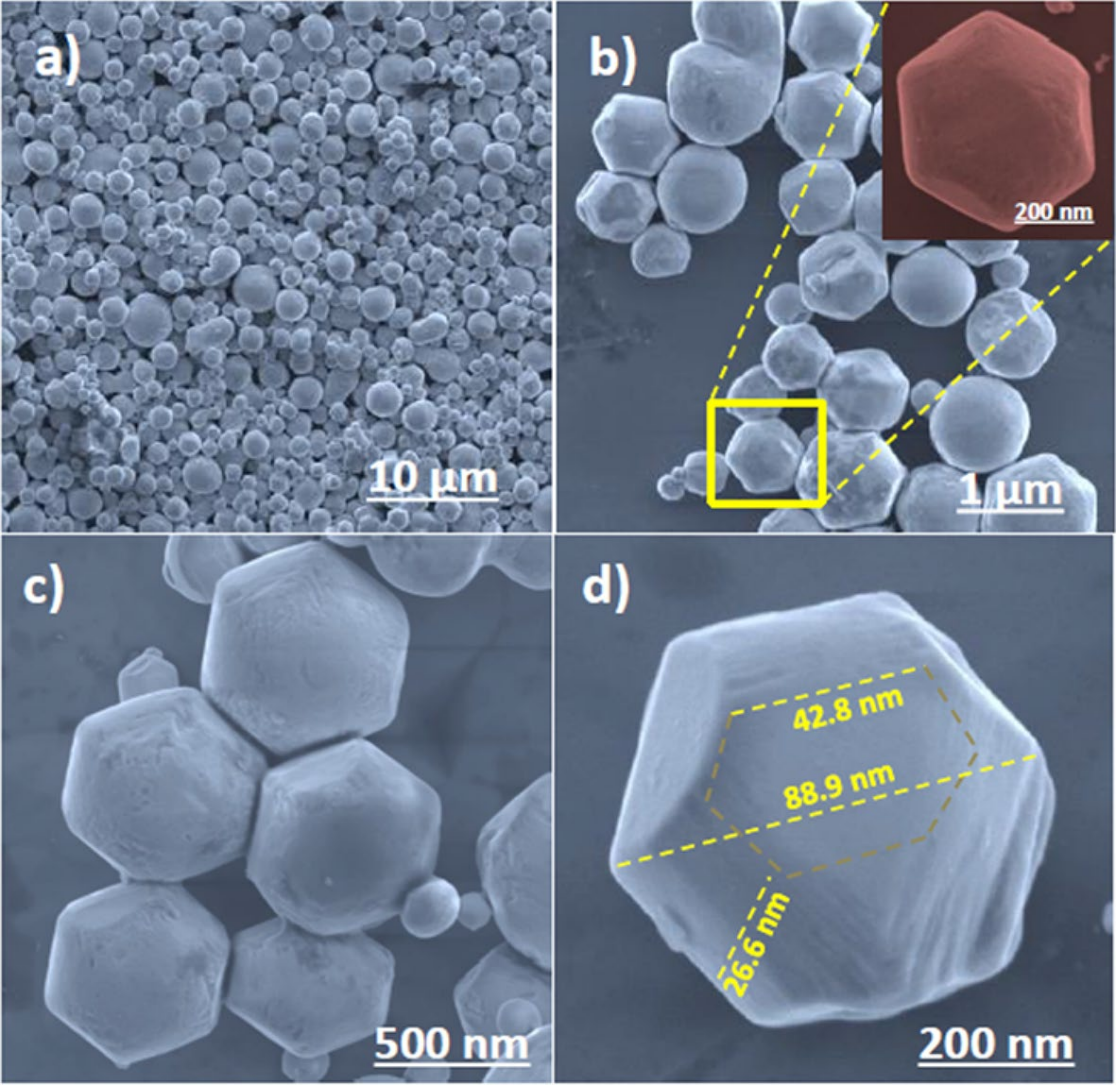
FE-SEM images of pure m-BiVO_4_ photocatalyst (**a**) at 10 µm scale bar, (**b**) at 1 µm scale bar Inset at 200 µm scale bar showing the hexagonal structure of a single NP, (**c**) at 500 µm scale bar showing uniformly neighbouring NPs and (**d**) at 200 µm scale bar showing the rough diameter and thickness of a single NP.

The field emission scanning electron microscopy (FESEM) is used to examine the morphology and elemental composition of the m-BiVO_4_ under different magnification. A broad view of the m-BiVO_4_ batch (Fig. 4a, 10 µm scale bar) reveals different size and shape m-BiVO_4_ nanoparticles (NPs). A more detailed examination of several neighbouring m-BiVO_4_ NPs (Fig. 4b, 1 µm scale bar) shows many hexagonal and few sphere NPs. A closer inspection of a single hexagonal m-BiVO_4_ NP (inset in Fig. 4b, 200 nm scale bar) finds the appearance of non-planar surface. Higher magnifications (Fig. 4c, 500 nm scale bar) confirm its smooth surface structures with mostly truncated square (18 sided) hexagonal bipyramidal shape with exposed (040) facets (Fig. 4d, 200 nm scale bar). Detailed morphology information is further confirmed using high resolution transmission microscopy (HRTEM) from the top view (Supplementary Fig. S1a,b) and side view (Supplementary Fig. S1c,d). Photocatalysis is a surface phenomenon and the facet effect strongly relates to formation of surface-active sites. The formation of different truncated and decagonal morphologies with (040) surface facets^49^ have found that facet surface energy played a very important role in determining the photocatalytic activity. The m-BiVO_4_ NP (Fig. 4c) shows rough diameter approximately 50–120 nm and thickness approximately 20–50 nm, leading to higher active surface area and enhancing its photocatalytic activities.

**Figure 5:**
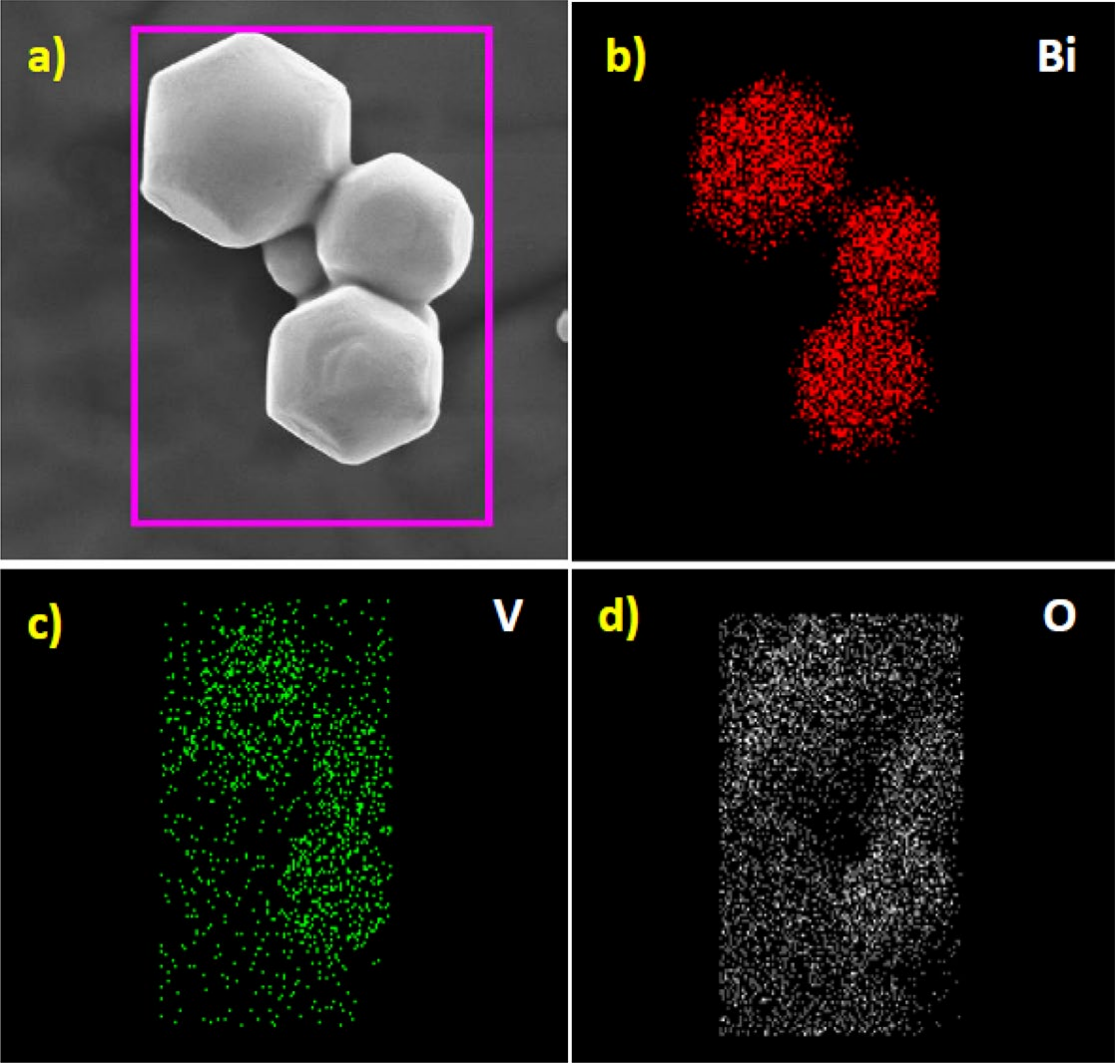
SEM elemental mapping image of (**a**) m-BiVO_4_ photocatalyst showing the presence of (**b**) Bi, (**c**) V and (**d**) O.

The elemental mapping composition of m-BiVO_4_ was further studied by the energy-dispersive X-ray spectroscopy (EDS) attached on the FESEM. The spatial distribution of bismuth (Fig. 5b), vanadium (Fig. 5c) and oxygen (Fig. 5d) correlates well with the FESEM of m-BiVO_4_ (Fig. 5a) indicate they are uniformly distributed within the m-BiVO_4_. Furthermore, the EDS spectrum (Supplementary Fig. S2) shows strong signal of Bi, V and O elements which all embed well within the selected areas of m-BiVO_4_. The absence of other noticeable peaks in the EDS spectrum implies that the m-BiVO_4_ surface is free of impurities, presumable due to intrinsic template-free and surfactant-free advantages in solvothermal method. The EDS analysis results find the element wt% content in m-BiVO_4_ are Bi 72.56%, V 11.04% and O 16.40%, in accordance well with the preparation proportion of 1:1 as Bi to V ratio.

More detailed morphology information is investigated by HRTEM. The HRTEM (Supplementary information Fig. S3a) clearly exposes the uniform lattice fringes of the m-BiVO_4_ with an interplanar lattice spacing of 0.304 nm which resembles to the (121) crystallographic planes of m-BiVO_4_. The selected area electron diffraction (SAED) pattern of m-BiVO_4_ (Supplementary information Fig. S3b) coincides with the (121) crystallographic plane of monoclinic m-BiVO_4_.

### Photocatalytic activity and stability

**Figure 6:**
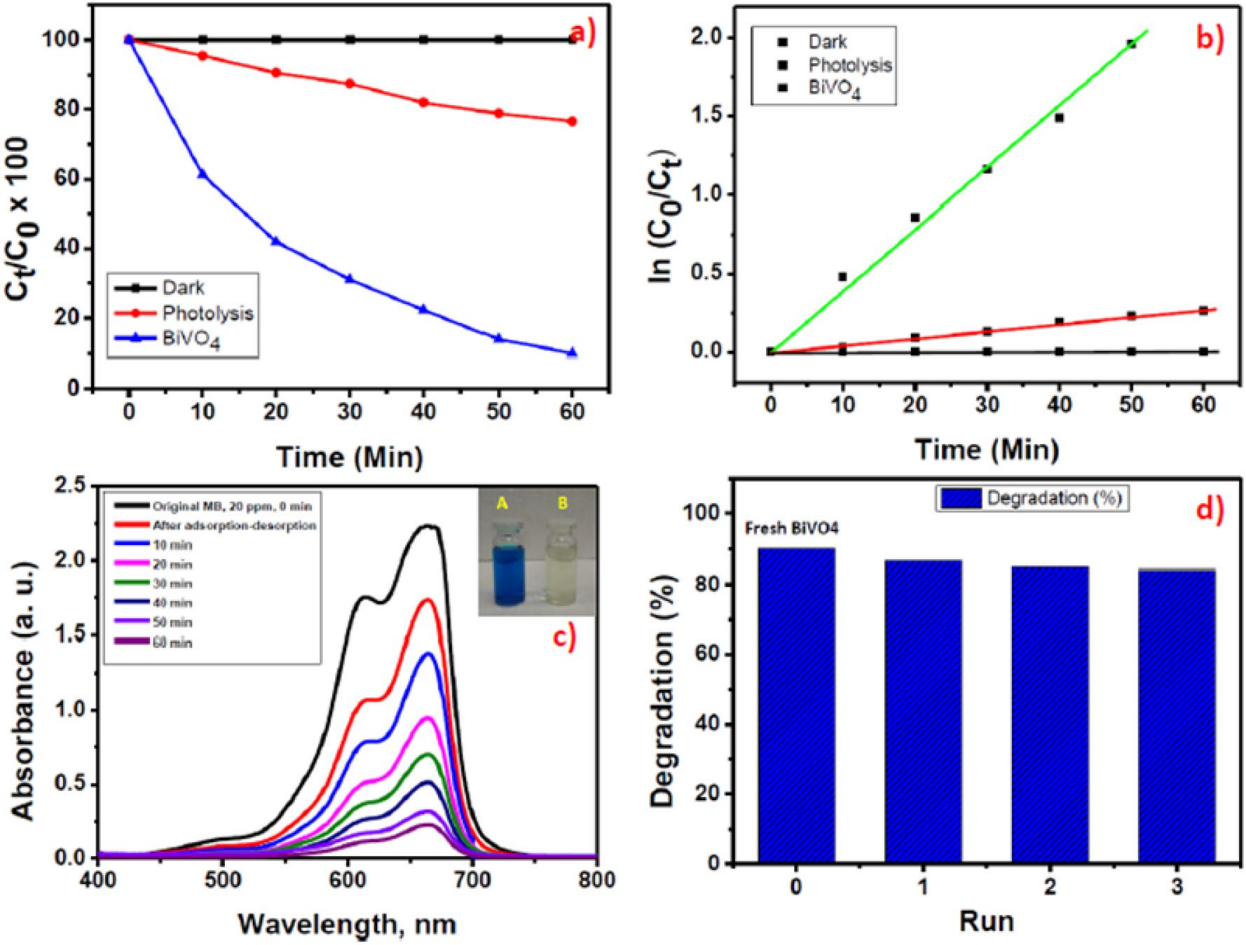
(**a**) Time-evolved photocatalytic degradation of MB under visible light irradiation, (**b**) Rate of chemical kinetics of the m-BiVO_4_ over photocatalytic MB degradation, (**c**) UV–Vis absorption spectra of the MB dye degradation by the recycled BiVO_4_ at successive time intervals during photocatalysis (Inset, photograph of the (A) 20 ppm MB solution and (B) 20 ppm MB treated solution), (**d**) Bar diagram of recycled runs of m-BiVO_4_ photocatalyst showing the MB dye degradation efficiency.

**Figure 7:**
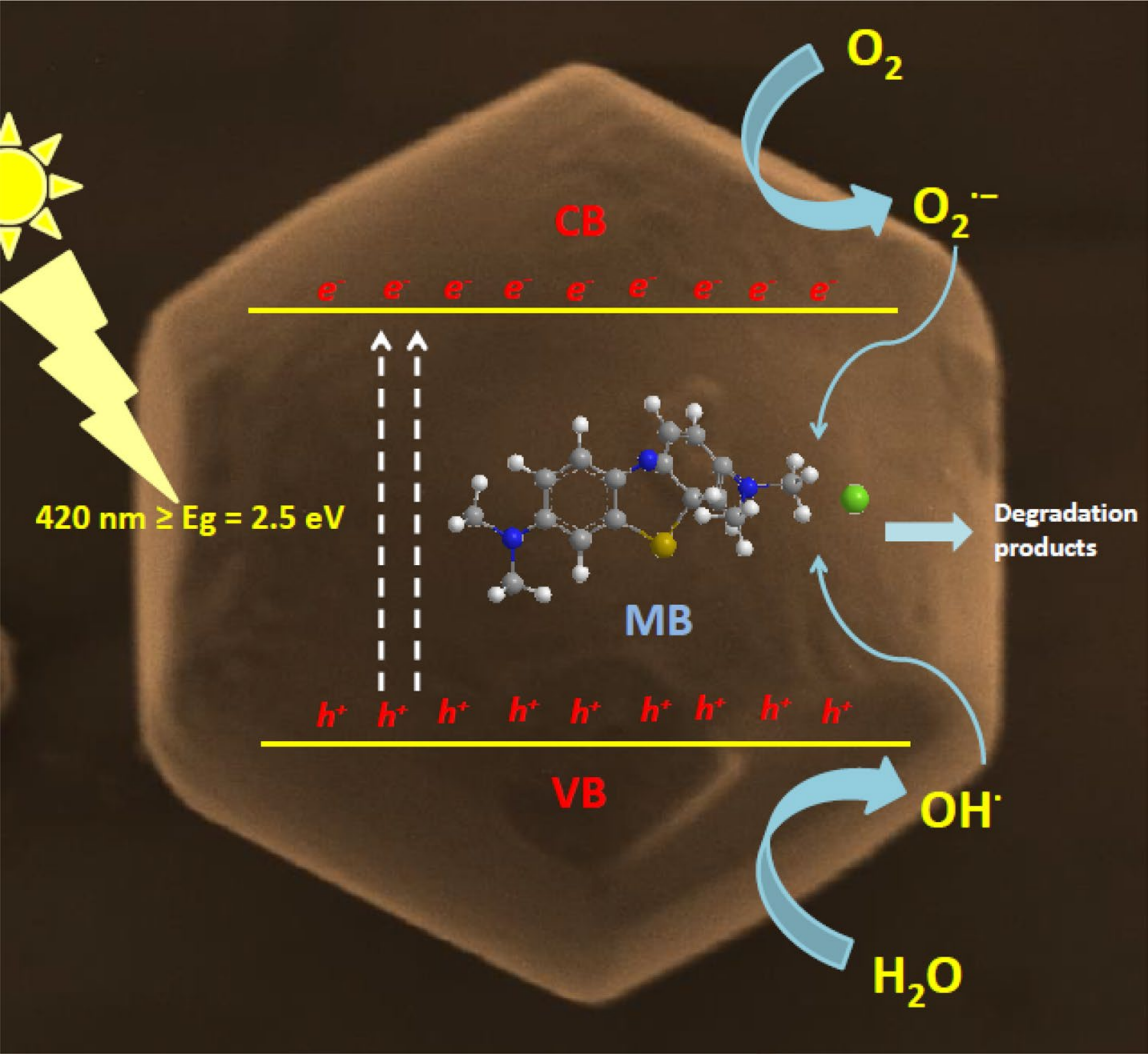
Schematic of MB photocatalytic degradation of m-BiVO_4_ photocatalyst (Truncated square, 18 sided) bipyramidal shape photocatalyst under visible light irradiation.

The photocatalytic activity of m-BiVO_4_ was investigated by degradation of MB irradiated with visible light (λ > 420 nm). MB is an odourless and dark blue-black heterocyclic aromatic compound. It forms a blue coloured solution when dissolved in room temperature water. Most textile plants use MB for dyeing purpose. MB is highly soluble in water and is very difficult to dispose before discharge into environment. Trace amounts of MB are still harmful to humans as well as aquatic life^6^. Figure 6a shows the photocatalytic MB degradation efficiency in the absence and presence of m-BiVO_4_ photocatalyst. Under dark condition, only negligible amounts of MB were degraded, presumably due to the adsorption of dye onto the surface of the catalyst. In contrast to the dark condition, the degradation activity was extremely enhanced and achieved about 91% degradation efficiency under light irradiation condition in the presence of m-BiVO_4_ photocatalyst in 60 min, presumably due to the presence of high-active (040) facets^41,42^. The rate constant of the photocatalytic reaction (Fig. 6b) is calculated from the slope of the plot of *ln*(C_o_/C_t_) versus irradiation time, where C_o_ is the initial MB concentration and C_t_ is the concentration of MB at the time t. The rate constant k is 0.388 × 10^−2^ min^−1^, indicating that the m-BiVO_4_ photocatalyst improved its degradation on MB dyes when exposed to visible light. The UV–Vis spectrum of 20 ppm MB solution after different times of VLD photocatalytic reaction (Fig. 6c) shows corresponding absorbance decrease and the colour changes from initial blue to final transparent (Inset in Fig. 6c). In practical applications, the stability of the photocatalyst is very important. Continuously recycle experiments show the reusability potential of m-BiVO_4_ for MB treatment (Fig. 6d). During the first treatment, most of the surface active sites were occupied by the MB. As the treatment was continued, the number of active sites available for the MB subsequently decreased, and as a result, the degradation efficiency of MB would be decreased. After three consecutive cycles, no noticeable deactivation of m-BiVO_4_ was observed. The results of the recycle experiments (Fig. 6d) indicate that the m-BiVO_4_ photocatalyst exhibits a reliability of activity and good stability with excellent degradation efficiency for MB. Under visible light irradiation, the electrons in m-BiVO_4_ generated from VB to CB because of their small band gap (Eg = 2.5 eV). Moreover, due to the different energy levels in BiVO_4_ (040) and BiVO_4_ (110) facets, we presume that the separation of electron–hole pairs among the BiVO_4_ (040) and BiVO_4_ (110) facets may enlarge the potential differences. The reduction and the oxidation reactions may therefore preferentially happen separately on the BiVO_4_ (040) and BiVO_4_ (110) facets surface. The results indicate that with rational structure design of facet-dependent BiVO_4_ semiconductor with small band gap can achieve faster degradation rate^43^. To assess the active species generated in the dye degradation of MB over BiVO_4_, the trapping experiment was performed. In species capturing process, various scavengers such as ethylene diamine tetraacetic acid disodium salt (EDTA-2Na), 1,4-benzoquinone (BQ) and isopropyl alcohol (IPA) was added into the reaction solution as a quenchers of holes (h^+^), superoxide radicals (O_2_^•−^) and hydroxyl radicals (^•^OH), respectively^44,45^. The degradation efficiency of MB was significantly inhibited by the addition of EDTA-2Na and slightly suppressed by the addition of BQ and IPA (Supplementary Fig. S4a). The addition of scavengers resulted in decreased degradation is also evidenced by the decreased rate constant (Supplementary Fig. S4b) from k = 0.38 × 10^−2^ min^−1^ (without scavenger) to k = 0.21 × 10^−2^ min^−1^ (IPA), 0.14 × 10^−2^ min^−1^ (BQ) and k = 0.02 × 10^−2^ min^−1^ (EDTA-2Na). The results indicate that a large number of holes (h^+^) in the VB of m-BiVO_4_ have offers powerful oxidation ability and plays as the main active species in the MB degradation. Based on the active species trapping analysis and band energy level analysis results, we propose a schematic of photocatalytic degradation of MB by VLD m-BiVO_4_ (Fig. 7), mainly due to the transfer behaviour of photo-generated electrons and holes among the m-BiVO_4_ (040) facet and the follow up reactions between the generated active species and B and BiVO_4_. In-depth mechanistic insights are available elsewhere (Supplementary Discussion S1). In brief, h^+^_VB_ is the key active species for the degradation of MB by VLD m-BiVO_4_ and the O^−2•^ and OH^•^ radicles play supplementary roles during the MB degradation.

### Zebrafish embryo toxicity test of the raw and treated MB dye solutions

**Figure 8:**
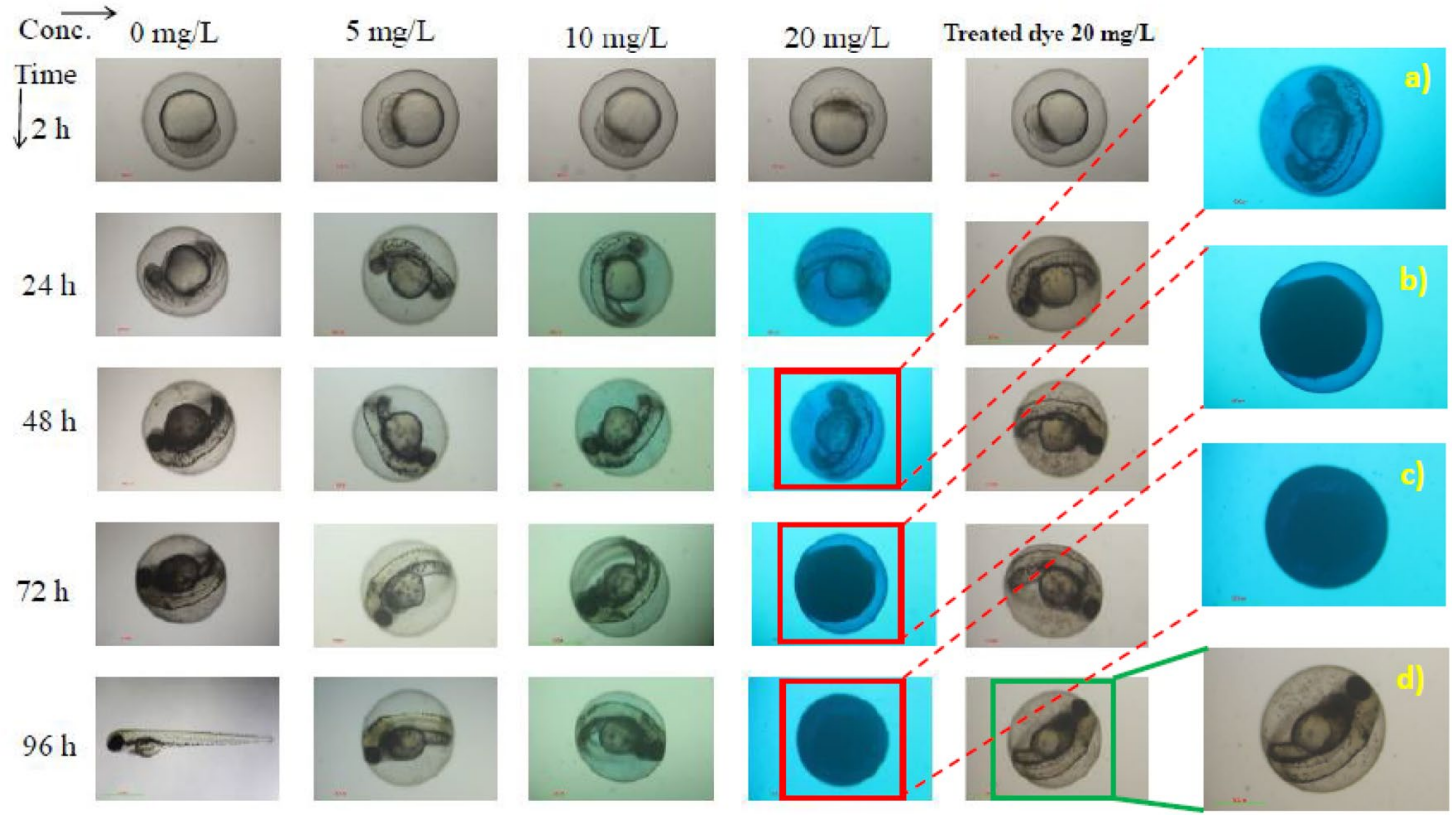
Toxicity effect of MB and treated MB solutions on Zebrafish embryo in different exposure of time and concentration of MB. (**a**–**c**) showing the damage to Zebrafish embryo after 48 hpf, 72 hpf and 96 hpf, respectively, in 20 ppm MB solution. (**d**) showing unaffected Zebrafish embryo in treated 20 ppm MB solution even after 96 hpf.

In photocatalytically dye degradation process, smaller degradation fragments are produced as by-products from the breaking of dye molecules which sometimes are even more toxic than the parent dyes. It is therefore obligatory to investigate the subsistence capacity of the living organism and its biological response after dye degradation^46^. We therefore investigate the ecotoxicity of the raw and photocatalytically treated MB dye solutions by Zebrafish embryo toxicity test^47,48^. On the potential for chemical stressors to affect ecosystems. Herein, the test is based on a 24 h exposure of 2, 24, 48, 72 and 96 h post fertilization (hpf) embryos in a static system. The rates of morphological changes as well as the survival capability are used as endpoints used to generate dose response curves. The Zebrafish embryo is an ideal model as its internal structures are nearly transparent and genetic structure are almost similar to humans. The rates of morphological change results show that the Zebrafish embryo was highly affected in raw MB solution, i.e. 72 hpf (Inset c in Fig. 8) and 96 hpf (Inset d in Fig. 8); whereas the VLD m-BiVO_4_ treated MB solution (20 ppm) was little affected, i.e. up to 96 hpf (Inset d in Fig. 8). Furthermore, it was noticed that as increased MB concentration (5 to 20 ppm) along with exposure time the MB toxicity expressed as mortality, teratogenicity and survivability apparently worsened on the Zebrafish embryo (Supplementary Table S2). The zebrafish embryo toxicity test results demonstrate that the raw (untreated) MB dye solution is toxic for aquatic life. After treatment by VLD m-BiVO_4_ photocatalyst, the toxicity considerably decreased to a tolerance level. Obviously, the presence of inorganic ions such as iron, zinc, magnesium, calcium, copper, bicarbonate, nitrate, sulphate, phosphate and chlorides in natural water and polluted water might eventually be adsorbed onto the surface of photocatalyst and retard the rate of photocatalytic reaction^49,50^.

## Methods

### Chemical reagents and materials

All reagents and chemicals used are of analytical grade. Sodium metavanadate (Na_3_VO_4_, Alfa Aesar), bismuth nitrate (Bi(NO_3_)_3_·5H_2_O, Showa Chemicals), methylene blue (Sigma Aldrich), ethylene glycol ((CH_2_OH)_2_, Merck) are of analytical reagent grade and used without further purification. Deionized water is used throughout in all experiments.

### Synthesis m-BiVO_4_

The solvothermal synthesis of m-BiVO_4_ was carried out by mixing corresponding amount of Bi(NO_3_)_3_·5H_2_O and NH_4_VO_3_, namely, Bi(NO_3_)_3_·5H_2_O (0.197 g) dissolved in ethylene glycol (20 mL) with a 1:1 stoichiometric amount of NH_4_VO_3_ (0.060 g) dissolved in ethylene glycol (20 mL). These two ethylene glycol solutions were ultrasonicated for 10 min first, then mixed to form a yellow brownish suspension which is kept under stirring for 1 h at room temperature. The resulting suspension was transferred into a Teflon reactor (100 mL) fitted into a stainless-steel autoclave and kept in an oven at 250 °C for 24 h. The solvothermal reaction was performed at 250 °C for 24 h. The Teflon reactor was allowed to cool naturally to room temperature. The as-obtained precipitate collected from the Teflon reactor was washed repeatedly with distilled water and ethanol follow by drying overnight at 60 °C. The thus-obtained as-synthesized m-BiVO_4_ samples were used for further characterization and application.

### Materials characterization

The crystalline structure of the as-synthesized m-BiVO_4_ was determined by X-ray diffraction (XRD) pattern using a X-ray diffraction spectrometer (Bruker AXS D2 Phaser) with a CuKα target (λ = 0.15418 nm, 40 kV and 100 mA) in the 2ϴ range of 10–80° at a scan rate of 1°/min. Laser Raman spectra were recorded using a Raman spectrometer (JOBIN YVON LabRam HR S8000U) excited with the 632.8 nm line of red laser at an 10 mW incident power. Optical absorption spectra were recorded using a UV–Vis spectrometer (Hitachi U-3300). The X-ray photoelectron spectra (XPS) were recorded on a XPS spectrometer (HRXPS-PHI Quantera SXM, Φ ULVAC-PHI, INC) at room temperature. The binding energy reference was taken at 284.7 eV for the C1s peak arising from the surface hydrocarbons. Prior to SEM analysis, the m-BiVO_4_ NPs were dispersed in ethanol and small drops of suspended solution were kept on a Si wafer piece and dried in vacuum oven. The surface morphology and sample composition were determined using a field emission scanning electron microscope (FE-SEM; JEOL JSM-7000F) attached with an energy dispersive X-ray spectroscopy analyser (EDS; OXFORD Instruments, INCA PentaFETx3, Model 7,557). Prior to TEM analysis, the m-BiVO_4_ NPs were dispersed in ethanol using an ultrasonicator and dropped onto a copper grid and dried in vacuum oven. The transmission electron microscopy (TEM). high-resolution transmission electron microscopy (HRTEM) and selected area electron diffraction (SAED) patterns were recorded using a JEOL JEM-2100 with an accelerating voltage of 80 kV and 200 kV, respectively. The time-evolved photocatalytic degradation of MB was measured by a T60 UV–Visible spectrophotometer (PG INSTRUMENTS LIMITED). The toxicity of raw and treated MB solutions were tested by Zebrafish embryo, the images of Zebrafish embryo were captured by a high resolution microscope (OLYMPUS 1X71, Lenses EW 10 x /22) attached with a Canon camera (EOS 650D).

### Photocatalytic dye degradation

Methylene blue with a major absorption band at 668 nm was used as a model pollutant for evaluation of photocatalytic activity of m-BiVO_4_. The experiment was carried out under visible light irradiation at room temperature. Photo-irradiation was carried out using a 1000 W xenon lamp (λ ≥ 420 nm) with a UV cut-off filter to deliver only visible radiation below 420 nm. The distance between the lamp and the MB solution was 20 cm. The photocatalytic degradation of the MB (20 ppm) dye was performed in an aqueous solution. Appropriate amount of m-BiVO_4_ (10 mg) was dispersed in an aqueous solution containing MB. Before starting irradiation, the reaction mixture was sonicated for half hour and further stirred for another half hour all in the darkness to reach adsorption − desorption equilibrium between the MB dye and the m-BiVO_4_ photocatalyst. The absorbance of MB for adsorption reaction was measured first followed by visible light irradiation under constant magnetic stirring. An aliquot (5 mL) was drawn at regular intervals (10 min) and centrifuged (1,000 rpm) to remove photocatalyst NPs. Different dye concentrations (blank, 5, 10 and 20 ppm) were prepared and studied. The photocatalytic degradation efficiency of the dye was estimated with C_t_/C_0_, where C_t_ was the concentration of dye at each irradiated time interval, and C_0_ was the initial concentration of the dye, respectively. At each irradiated time interval, the UV–Vis absorbance spectrum of treated solution was measured using a UV–Vis spectrophotometer under full scan mode.

### Reactive species capturing experiment

The active species generated during the photocatalytic reaction play very important role. In situ reactive species capture experiments were conducted to identify possible active species by the addition of trapping agents (scavengers). The scavengers such as disodium ethylene diamine tetraacetate (EDTA-2Na), 1,4-benzoquinone (BQ), and isopropyl alcohol (IPA) were added as a quencher for capturing the holes (h^+^), superoxide radicals (O2^•**−**^) and hydroxyl radicals (^•^OH), respectively. The EDTA (0.1 mol L^−1^), benzoquinone (0.1 mol L^−1^) and isopropyl alcohol (0.1 mol L^−1^) was separately added (10 mL) in each experiment into a MB solution^40^.

### Assessment of dye toxicity on zebrafish embryo and experimental setup

The ecotoxicity of industrial effluent is important which could be verified by the corresponding water quality and survival capability of inherent aquatic animal species. We follow the Taiwan EPA standard method, namely, Method for Testing the Biological Acute Toxicity-Zebrafish Embryo in Semi-Static Water^47^ which is based on the OECD Test No. 236: Fish embryo acute toxicity (FET) test. Guidelines for the Testing of Chemicals^48^. The temperature of artificial tank was maintained at 27 °C. The toxicity of MB and treated dye solution were carried out on the newly hatched zebrafish embryo. The fresh zebrafish embryos were collected at room temperature and added into different concentration of MB solution (5, 10 and 20 ppm). The affected organs of embryo were inspected under a high-resolution microscope attached by camera after 2, 24, 48, 72, and 96 hpf interval. The experiments were carried out in triplicate for each MB solution and the same toxicity tests were performed in the treated MB solution (i.e. dye degradation solution). Zebrafish are handled according to guidelines and regulations of Laboratory Animal Care and Use Committee (Permission No. 10314), National Tsing Hua University, Taiwan. The study was approved by the same.

## Supplementary information

Supplementary Information.
