# Supplementary material for: Solvothermal synthesis of facet-dependent BiVO4 photocatalyst with enhanced visible-light-driven photocatalytic degradation of organic pollutant: assessment of toxicity by zebrafish embryo
Source: Sci Rep. 2020 Aug 3;10:12993. doi: 10.1038/s41598-020-69706-4 (PMC7398900; doi:10.1038/s41598-020-69706-4)
Supplement: Supplementary file 1 — Supplementary Information. [file 41598_2020_69706_MOESM1_ESM.docx]

**Supplementary information**

**Solvothermal Synthesis of Facet-dependent BiVO_4_ Photocatalyst for Enhanced Visible-light-driven Photocatalytic degradation of Organic Pollutant: Assessment of Toxicity by Zebrafish Embryo**

Ganesh S. Kamble^1,2^* and Yong-Chien Ling^1,3^*

^1^Department of Chemistry, National Tsing Hua University, Hsinchu 30013, Taiwan

^2^Department of Engineering Chemistry, Kolhapur Institute of Technology’s College of Engineering (Autonomous), Kolhapur 416234, India

^3^Institute of Nano Engineering and Micro Systems, National Tsing Hua University, Hsinchu 30013, Taiwan

*Corresponding Author: E-mail: [ycling@mx.nthu.edu.tw](mailto:ycling@mx.nthu.edu.tw) (Y.C.L.)

[ganeshchemistry2010@gmail.com](mailto:ganeshchemistry2010@gmail.com) (G.S.K.)

**Introduction S1: Hydrothermal synthesis of BiVO_4_ owing to various shapes and composites.**

In the recent hydrothermal synthesis of BiVO_4_, countless development has been achieved on its shape structures such as microtubular spindle,^1^ microspherical,^2^ nanosheet-like,^3^ hyperbranched,^4^ nanoribbon-like,^5^ nanoplate-like,^6^ hollow shell-like,^7^ and cocoon-like.^8^ However, the development of a surfactant-free, toxic chemical-free and facile hydrothermal method for the synthesis of BiVO_4_ with desired structure, surface area, morphology, and stability as affordable VLD photocatalyst with enhanced photocatalytic activities is still mandated. Several research groups have addressed this challenge and synthesized novel BiVO_4_ photocatalysts for VLD degradation of aqueous organic dye i.e. methylene blue (MB) with enhanced photocatalytic activity such as doped BiVO_4_ and composite BiVO_4_ including Au/BiVO_4_,^9^ rGO-BiVO_4_,^10^ Cu/BiVO_4_,^11^ rGO/Ag/BiVO_4_,^12,13^ and Er/BiVO_4_^14-16^ In addition, heterojunction, Z-scheme photocatalysts such as Cu_2_O/BiVO_4_,^17^ WO_3_/BiVO_4_,^18^ GeO_2_/BiVO_4_,^19^ CeO_2_/BiVO_4_,^20^ Bi_2_O_3_/BiVO_4_,^21,22^ V_2_O_5_/BiVO_4_,^23^ BiVO_4_/TiO_2_,^24^ Ag_3_PO_4_/BiVO_4_,^25,26^ Co_3_O_4_/BiVO_4_,^27^ BiOCl/BiVO_4_,^28^ CdS–Au–BiVO_4_,^29^ Titanosilicate BiVO_4_,^30^ BiIO_4_/BiVO_4_,^31^ etc. The aforementioned methods still allow for further improvements which have mentioned in the Table S1.

**Table S1. Comparison of the present work with other synthesized method of BiVO_4_ and photocatalytic dye degradation performance**

| **Sr. No.** | **Photocatalyst** | **Photocatalyst conc.,**  **Mg** | **Organic model pollutant** | **Conc. of organic pollutant/**  **model,**  **Mg L^-1^** | **Degradation time, Min** | **Remarks/Drawbacks** | **Ref. No.** |
| --- | --- | --- | --- | --- | --- | --- | --- |
|  |  |  |  |  |  |  |  |
| 1 | BiVO_4_ | 200 | Rhodamine B | 10 | 300 | - Large quantity of catalyst concentration - More degradation time | [1] |
|  |  |  |  |  |  |  |  |
| 2 | BiVO_4_ | 200 | Methylene blue | 2.0 | 90 | - Large quantity of catalyst concentration - More degradation time | [50@] |
|  |  |  |  |  |  |  |  |
| 4 | BiVO_4_ | 150 | Methylene blue | 4.2 | 50 | - Large quantity of catalyst concentration - Use of citric acid surfactant and complicated synthesized steps of catalyst | [51@] |
|  |  |  |  |  |  |  |  |
| 5 | BiVO_4_ | 500 | *N,N,N’,N’*-tetraethylated rhodamine | 2.4 | 180 | - Large quantity of catalyst concentration - More degradation time - Use of citric acid surfactant and complicated synthesized steps of catalyst | [2] |
|  |  |  |  |  |  |  |  |
| 6 | BiVO_4_ | 200 | *N,N,N’,N’*-tetraethylated rhodamine | 5 | 45 | - Large quantity of catalyst concentration - Less conc. of organic pollutant - pH 2.0-3.0 | [3] |
|  |  |  |  |  |  |  |  |
| 7 | BiVO_4_ | 150 | Eosin Y | 5 | 40 | - Large quantity of catalyst concentration - Less conc. of organic pollutant | [4] |
|  |  |  |  |  |  |  |  |
| 8 | Cu/ BiVO_4_ | 30 | Rhodamine B | 15 | 50 | - Complicated synthesized steps of catalyst | [8] |
|  |  |  |  |  |  |  |  |
| 9 | Au- BiVO_4_ | 10 | Methyl Orange | 5 | 120 | - Use of 0.01 M L^-1^ cysteine and 0.05 M L^-1^ ascorbic acid as a surfactant and complicated synthesized steps of catalyst | [9] |
|  |  |  |  |  |  |  |  |
| 10 | rGO-BiVO_4_ | 100 | Methyl blue and Rhodamine B | 10 | 60 | - Large quantity of catalyst concentration - pH 6.0 | [10] |
|  |  |  |  |  |  |  |  |
| 11 | Cu/ BiVO_4_ | 50 | Rhodamine B | 5 | 210 | - Large quantity of catalyst concentration - More degradation time | [11] |
|  |  |  |  |  |  |  |  |
| 12 | rGo/Ag/ BiVO_4_ | 10 | Phenol, Methylene blue | 10 | 90 | - Large quantity of catalyst concentration - Involved more steps for synthesise of catalyst - Less rate of reaction *k* = 1.03 × 10^−2^ min^−1^) | [12] |
|  |  |  |  |  |  |  |  |
| 13 | Er/ BiVO_4_ | 50 | Methylene blue | 10 | 120 | - Large quantity of catalyst concentration - More degradation time - pH 9.0 | [16] |
|  |  |  |  |  |  |  |  |

| 14 | Cu_2_O/ BiVO_4_ | 30 | 4-chlorophenol | 100 | 240 | - Complicated synthesized steps of catalyst - More degradation time - Use of reducing agents such as 0.7 M of ascorbic acid and 1.4 M of fructose or glucose | [17] |
| --- | --- | --- | --- | --- | --- | --- | --- |
|  |  |  |  |  |  |  |  |
| 15 | WO_3_/ BiVO_4_ | Photoanode film | Tetracycline hydrochloride (TC), Phenol and Congo Red (CR) | 0.683 TC, 0.385 Phenol, 1.05 CR | 180 | - Complicated synthesized steps of catalyst - More degradation time | [18] |
| 16 | GeO_2_/ BiVO_4_ | 50 | Rhodamine B | 1.96 | 240 | - Complicated synthesized steps of catalyst - More degradation time | [19] |
|  |  |  |  |  |  |  |  |
| 17 | WO_3_, TiO_2_ or CeO_2_/ BiVO_4_ | 500 | Methylene blue (MB), Rhodamine B (RB) | MB~50, RB~25 | 360 | - Complicated synthesized steps of catalyst - Large quantity of catalyst concentration - More degradation time | [20] |
|  |  |  |  |  |  |  |  |
| 18 | GO/Bi_2_O_3_/ BiVO_4_ | 100 | Bisphenol-A | 10 | 300 | - Complicated synthesized steps of catalyst - Large quantity of catalyst concentration - More degradation time | [22] |
|  |  |  |  |  |  |  |  |
| 19 | V_2_O_5_/ BiVO_4_/TiO_2_ | 30 | Tolune | 4 | 360 | - Complicated synthesized steps of catalyst - More degradation time | [23] |
|  |  |  |  |  |  |  |  |
| 20 | BiVO_4_-TiO_2_ SILAR | Photoanode film | Rhodamine 6G | 6 | 300 | - Complicated synthesized steps of catalyst - More degradation time | [24] |
|  |  |  |  |  |  |  |  |
| 21 | Ag_3_PO_4_ /BiVO_4_ | 50 | Methylene blue | 10 | 30 | - Complicated synthesized steps of catalyst | [25] |
|  |  |  |  |  |  |  |  |
| 22 | Ag/Ag_3_PO_4_/BiVO_4_/RGO | 10 | Tetracycline | 10 | 60 | - Complicated synthesized steps of catalyst | [26] |
|  |  |  |  |  |  |  |  |
| 23 | Co_3_O_4_/BiVO_4_ | Not mentioned | Phenol | 55% | 180 | - Complicated synthesized steps of catalyst - More degradation time - 55% pollutant degraded | [27] |
|  |  |  |  |  |  |  |  |
| 24 | BiOCl/BiVO_4_ | 100 | Methyl Ornage | 2.63 | 650 | - Complicated synthesized steps of catalyst - 6 M NaOH - Large quantity of catalyst concentration - More irradiation time | [28] |
|  |  |  |  |  |  |  |  |
| 25 | CdS–Au–BiVO_4_ | 25 for TC 100 for RhB | Tetracycline (TC) and Rhodamine B (RhB) | 10 | 90 | - Complicated synthesized steps of catalyst - Use of reducing agent (0.2 M Oxalic Acid) | [29] |
|  |  |  |  |  |  |  |  |
| 26 | Titanosilicate BiVO_4_ | 100 | Rhodamine B | 5 | 60 | - Complicated synthesized steps of catalyst - Use of surfactant (0.05 M CTAB) | [30] |
|  |  |  |  |  |  |  |  |
| 27 | BiIO_4_/BiVO_4_ | 50 | Bisphenol A (BPA), Rhodamine B (RhB) | 10 (BPA),  1 (RhB) | 300 | - Complicated synthesized steps of catalyst - More irradiation time | [31] |
|  |  |  |  |  |  |  |  |

| 28 | m-BiVO_4_ (Truncated square (18 sided) hexagonal bipyramidal structure) | 10 | Methylene blue | 20 | 60 | - Single step synthesized catalyst - Less concentration of catalyst - Less irradiation time - Fast rate of reaction, *k* = 3.9 × 10^−2^ min^−1^ | P. M. |
| --- | --- | --- | --- | --- | --- | --- | --- |

@ = Main manuscript

*P. M. = Present Method

**S1: HRTEM images of m-BiVO_4_**


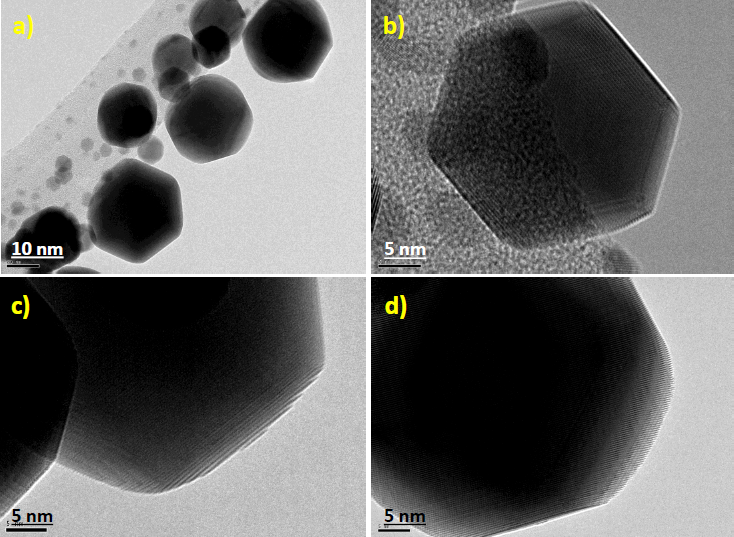


**Figure S1.** HRTEM images of m-BiVO_4_: Top view (a) at 10 nm scale bar, (b) at 5 nm scale bar, and Side view (c) at 5 nm scale bar, (d) at 5 nm scale bar.

**S2: EDS spectrum of m-BiVO_4_**


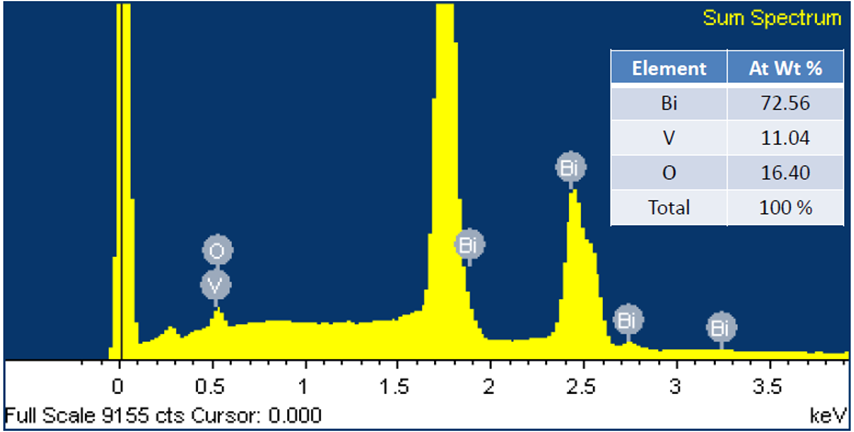


**Figure S2.** The EDS spectrum of m-BiVO_4_ and inset showing Bi, V, and O element content by wt%.

**S3: Lattice fringes and SAED pattern of m-BiVO_4_**


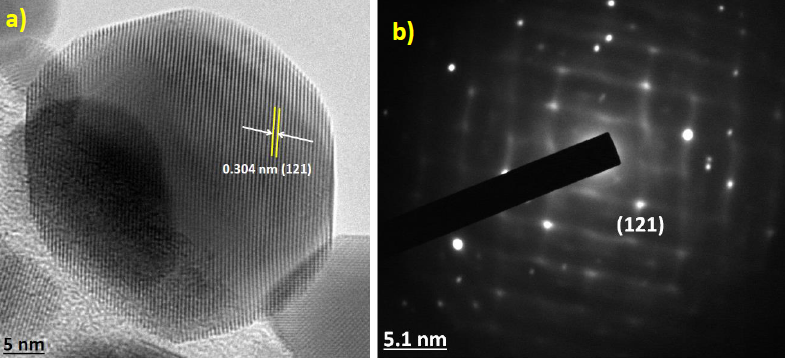


**Figure S3.** HRTEM (a) lattice fringes and (b) SAED pattern of m-BiVO_4_

**S4: Time-evolved photocatalytic degradation of MB using m-BiVO_4_ photocatalyst**


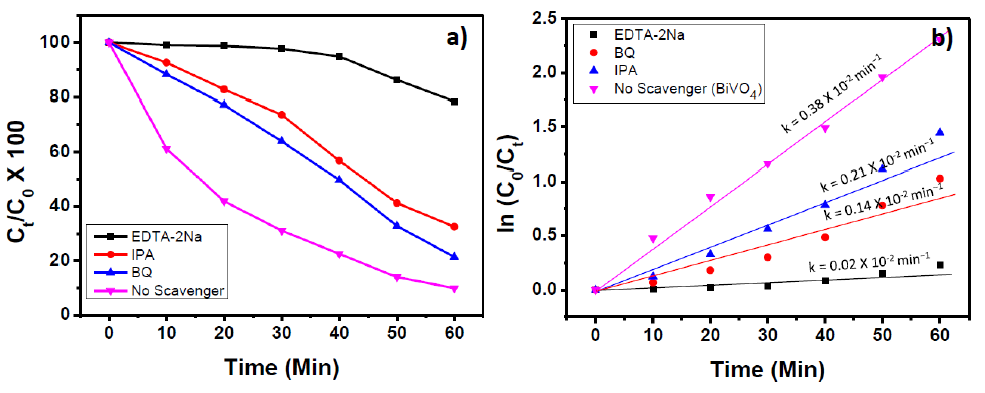


**Figure S4**. Time-evolved (a) Photocatalytic degradation of MB using BiVO_4_ photocatalyst under visible light irradiation in the presence of scavengers such as EDTA-2Na, IPA and BQ, (b) pseudo first order rate constants

**Discussion S1: MB** **degradation reaction mechanism.**

Under visible light irradiation, electrons from the valence band (VB) transported to the conduction band (CB) and formed electron–hole pairs. At the time, the electrons from the CB reacted with O_2_ to form O_2_^∙−^) which could also the degradation of MB under visible light irradiation. Commonly, in a semiconducting photocatalytic reaction, the potential of the valence band is more positive than the potential of the conduction band and vice versa. The potential of conduction band (CB) and valence band (VB) can be calculated according to the following equations (1) and (2) as follows:^32^

*E*_VB_ = *X* – *E*^e^ + 0.5 (*E*_g_) (1)

*E*_CB_ = *E*_VB_ − *E*_g_ (2)

where *E*_VB_ and *E*_CB_ are the potential of valence band and conduction band, respectively; *X* is the electronegativity of the semiconductor, expressed as the geometric mean of the electronegativity of the constituent atoms; *E*^e^ is the energy of free electrons on the hydrogen scale is about 4.5 eV, *E*g is the band gap energy of the semiconductor. Accordingly, the *E*_VB_ and *E*_CB_ of BiVO_4_ were separately estimated to be 3.40 ev and 0.9 eV, respectively.

Based on the above results from the active species trapping analysis and band energy level analysis, the possible photocatalytic mechanism is illustrated in the following Figure.


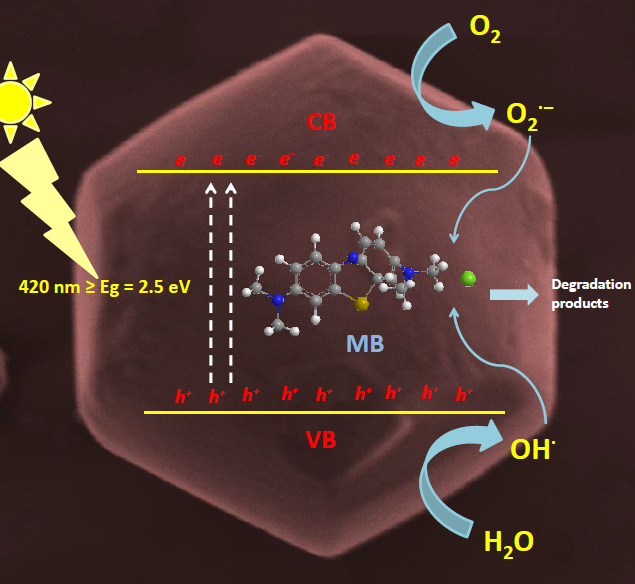


During the photocatalytic process, the active species of BiVO_4_ generated and possible reactions for degradation of MB have described in detail as follows. Under the visible light irradiation, the electrons from the valence band transited to the conduction band to form photogenerated hole–electron pairs (4). Electrons from CB reacted with O_2_ adsorbed on the surface of the BiVO_4_ to generate O_2_^•−^ (5). Simultaneously, OH^•^ radicals were formed by photoholes accepting electrons from OH^−^ (H_2_O) (6). Peroxide hydroxyl radical (HO^2•^) was produced through the protonation of O^−2•^ by reacting with H^+^ and disintegrated into hydrogen peroxide (H_2_O_2_) (7) and (8), which further oxidized with e^−^ produced OH^•^ (9). The same OH^•^ also consumed by MB for oxidation reaction to formed MB^•^ (10). According to active species trapping experiment, h^+^ direct reacts with MB which leading the MB degradation process (11).

The general photocatalysis degradation reaction mechanism of MB is summarized as following.^33,34^

1. Absorption of efficient visible light

Sunlight

MB MB*

*hν*

(420 nm ≥ Eg = 2.50 eV for BiVO_4_):

BiVO_4_ + *hν* → BiVO_4_ (e^−^_CB_ + h^+^_VB_) (4)

2. Formation of an oxygen ionosorption:

(O_2_)_ads_ + e^−^_CB_ → O_2_^•−^ (5)

3. Production of OH^•^ radicals by photoholes from H_2_O

(H_2_O H+ + OH^−^)_ads_ + h^+^_VB_ → H^+^ + OH^•^ (6)

4. Protonation of O^−2•^

O^−2•^ + H^+^ → HO^2•^ (7)

5. Hydrogen peroxide formation and dismutation of oxygen

2HO^2•^ → H_2_O_2_ + O_2_ (8)

6. Decomposition of H_2_O_2_

H_2_O_2_ + e^−^ → OH^•^ + OH^−^ (9)

7. Oxidation of the organic pollutant by OH• radicals

MB + OH^•^ → MB^•^ + H_2_O (10)

8. Direct oxidation reaction by holes

MB + h^+^_VB_ → MB^+•^ → degradation products (11)

Thus, the overall results indicate that, h^+^_VB_ is the key active species for the degradation of MB by BiVO_4_ under visible light irradiation and the O^−2•^ and OH^•^ radicles are played a supplementary role during the MB degradation, too.

MB* + (h^+^_VB_, OH^•^, O^−2•^, 2HO^2•^ and e^−^_CB_) Degraded products (H_2_O and CO_2_)

**Table S2. Toxicity effect of increased MB concentration on raw and VLD m-BiVO_4_ treated MB on Zebrafish embryo**

|  | MB dye Concentration (mg/L), 96 h | | | | |
| --- | --- | --- | --- | --- | --- |
|  | 0 | 5 | 10 | 20 | Treated Dye 20 |
|  |  |  |  |  |  |
| Mortality (%) | 0 | 0 | 0 | 50 | 0 |
| Teratogenicity (%) | 0 | 0 | 0 | 50 | 0 |
| survivability (%) | 100 | 100 | 50 | 30 | 70 |
|  |  | | | | |

**References:**

1. Liu, W. *et al.* Synthesis of monoclinic structured BiVO4 spindly microtubes in deep eutectic solvent and their application for dye degradation. *J Hazard Mater* **181**, 1102-1108, (2010).

2. Zhang, L., Chen, D. & Jiao, X. Monoclinic Structured BiVO4 Nanosheets:  Hydrothermal Preparation, Formation Mechanism, and Coloristic and Photocatalytic Properties. *The Journal of Physical Chemistry B* **110**, 2668-2673, (2006).

3. Zhao, Y., Xie, Y., Zhu, X., Yan, S. & Wang, S. Surfactant-Free Synthesis of Hyperbranched Monoclinic Bismuth Vanadate and its Applications in Photocatalysis, Gas Sensing, and Lithium-Ion Batteries. *Chemistry – A European Journal* **14**, 1601-1606, (2008).

4. Wang, F., Shao, M., Cheng, L., Hua, J. & Wei, X. The synthesis of monoclinic bismuth vanadate nanoribbons and studies of photoconductive, photoresponse, and photocatalytic properties. *Materials Research Bulletin* **44**, 1687-1691, (2009).

5. Neves, M. C. & Trindade, T. Chemical bath deposition of BiVO4. *Thin Solid Films* **406**, 93-97, (2002).

6. Sun, S., Wang, W., Zhou, L. & Xu, H. Efficient Methylene Blue Removal over Hydrothermally Synthesized Starlike BiVO4. *Industrial & Engineering Chemistry Research* **48**, 1735-1739, (2009).

7. Dunkle, S. S., Helmich, R. J. & Suslick, K. S. BiVO4 as a Visible-Light Photocatalyst Prepared by Ultrasonic Spray Pyrolysis. *The Journal of Physical Chemistry C* **113**, 11980-11983, (2009).

8. Wang, M. *et al.* Effects of Cu dopants on the structures and photocatalytic performance of cocoon-like Cu-BiVO4 prepared via ethylene glycol solvothermal method. *Journal of Alloys and Compounds* **691**, 8-14, (2017).

9. Cao, S.-W. *et al.* Preparation of Au-BiVO4 Heterogeneous Nanostructures as Highly Efficient Visible-Light Photocatalysts. *ACS Applied Materials & Interfaces* **4**, 418-423, (2012).

10. Wang, Y. *et al.* Electrostatic self-assembly of BiVO4-reduced graphene oxide nanocomposites for highly efficient visible light photocatalytic activities. *ACS Appl Mater Interfaces* **6**, 12698-12706, (2014).

11. Chen, P. A promising strategy to fabricate the Cu/BiVO4 photocatalysts and their enhanced visible-light-driven photocatalytic activities. *Journal of Materials Science: Materials in Electronics* **27**, 2394-2403, (2016).

12. Patil, S. S. *et al.* One-Pot in Situ Hydrothermal Growth of BiVO(4)/Ag/rGO Hybrid Architectures for Solar Water Splitting and Environmental Remediation. *Sci Rep* **7**, 8404, (2017).

13. Li, M. *et al.* Synthesis of Ag/BiVO4/rGO composite with enhanced photocatalytic degradation of triclosan. *Science of The Total Environment* **664**, 230-239, (2019).

14. Obregón, S. & Colón, G. Heterostructured Er3+ doped BiVO4 with exceptional photocatalytic performance by cooperative electronic and luminescence sensitization mechanism. *Applied Catalysis B: Environmental* **158-159**, 242-249, (2014).

15. Yang, W. *et al.* The upconversion and enhanced visible light photocatalytic activity of Er3+-doped tetragonal BiVO4. *RSC Advances* **5**, 7324-7329, (2015).

16. Obregón, S., Lee, S. W. & Colón, G. Exalted photocatalytic activity of tetragonal BiVO4 by Er3+ doping through a luminescence cooperative mechanism. *Dalton Transactions* **43**, 311-316, (2014).

17. Aguilera-Ruiz, E. *et al.* Facile synthesis of visible-light-driven Cu2O/BiVO4 composites for the photomineralization of recalcitrant pesticides. *RSC Advances* **7**, 45885-45895, (2017).

18. Zeng, Q. *et al.* Synthesis of WO3/BiVO4 photoanode using a reaction of bismuth nitrate with peroxovanadate on WO3 film for efficient photoelectrocatalytic water splitting and organic pollutant degradation. *Applied Catalysis B: Environmental* **217**, 21-29, (2017).

19. Natarajan, K., Bajaj, H. C. & Tayade, R. J. Direct sunlight driven photocatalytic activity of GeO2/monoclinic-BiVO4 nanoplate composites. *Solar Energy* **148**, 87-97, (2017).

20. Pingmuang, K. *et al.* Composite Photocatalysts Containing BiVO(4) for Degradation of Cationic Dyes. *Sci Rep* **7**, 8929, (2017).

21. Lopes, O. F., Carvalho, K. T. G., Avansi, W. & Ribeiro, C. Growth of BiVO4 Nanoparticles on a Bi2O3 Surface: Effect of Heterojunction Formation on Visible Irradiation-Driven Catalytic Performance. *The Journal of Physical Chemistry C* **121**, 13747-13756, (2017).

22. Qiu, P. *et al.* BiVO4/Bi2O3 heterojunction deposited on graphene for an enhanced visible-light photocatalytic activity. *Journal of Alloys and Compounds* **706**, 7-15, (2017).

23. Sun, J., Li, X., Zhao, Q., Ke, J. & Zhang, D. Novel V2O5/BiVO4/TiO2 Nanocomposites with High Visible-Light-Induced Photocatalytic Activity for the Degradation of Toluene. *The Journal of Physical Chemistry C* **118**, 10113-10121, (2014).

24. Odling, G. & Robertson, N. BiVO4 -TiO2 Composite Photocatalysts for Dye Degradation Formed Using the SILAR Method. *Chemphyschem* **17**, 2872-2880, (2016).

25. Li, C. *et al.* Selective deposition of Ag₃PO₄ on monoclinic BiVO₄(040) for highly efficient photocatalysis. *Small* **9**, 3951-3956, 3950, (2013).

26. Chen, F. *et al.* Hierarchical assembly of graphene-bridged Ag3PO4/Ag/BiVO4 (040) Z-scheme photocatalyst: An efficient, sustainable and heterogeneous catalyst with enhanced visible-light photoactivity towards tetracycline degradation under visible light irradiation. *Applied Catalysis B: Environmental* **200**, 330-342, (2017).

27. Long, M. *et al.* Efficient Photocatalytic Degradation of Phenol over Co3O4/BiVO4 Composite under Visible Light Irradiation. *The Journal of Physical Chemistry B* **110**, 20211-20216, (2006).

28. He, Z. *et al.* BiOCl/BiVO4 p–n Heterojunction with Enhanced Photocatalytic Activity under Visible-Light Irradiation. *The Journal of Physical Chemistry C* **118**, 389-398, (2014).

29. Bao, S., Wu, Q., Chang, S., Tian, B. & Zhang, J. Z-scheme CdS–Au–BiVO4 with enhanced photocatalytic activity for organic contaminant decomposition. *Catalysis Science & Technology* **7**, 124-132, (2017).

30. Adepu, A. K., Katta, V. & Narayanan, V. Synthesis, characterization, and photocatalytic degradation of Rhodamine B dye under sunlight irradiation of porous titanosilicate (TS)/bismuth vanadate (BiVO4) nanocomposite hybrid catalyst. *New Journal of Chemistry* **41**, 2498-2504, (2017).

31. Huang, H., Liu, L., Zhang, Y. & Tian, N. Novel BiIO4/BiVO4 composite photocatalyst with highly improved visible-light-induced photocatalytic performance for rhodamine B degradation and photocurrent generation. *RSC Advances* **5**, 1161-1167, (2015).

32. Sun, J., Li, X., Zhao, Q., Ke, J. & Zhang, D. Novel V_2_O_5_/BiVO_4_/TiO_2_ Nanocomposites

with High Visible-Light-Induced Photocatalytic Activity for the Degradation of

Toluene. *The Journal of Physical Chemistry C* **118**, 10113-10121 (2014).

33. Pingmuang, K. *et al.* Composite Photocatalysts Containing BiVO_4_ for Degradation of

Cationic Dyes. *Scientific reports* **7**, 8929 (2017).

34. Azeez, F. *et al.* The effect of surface charge on photocatalytic degradation of methylene

blue dye using chargeable titania nanoparticles. *Sci Rep* **8**, 7104 (2018).
